# Physical Activity, Body Composition, Serum Myokines and the Risk of Death in Hemodialysis Patients

**DOI:** 10.3390/medicina59112020

**Published:** 2023-11-16

**Authors:** Martyna A. Koźma-Śmiechowicz, Bartosz Gajewski, Paweł Fortak, Katarzyna Gajewska, Michał Nowicki

**Affiliations:** Department of Nephrology, Hypertension and Kidney Transplantation, Central University Hospital of the Medical University of Lodz, 90-222 Lodz, Poland

**Keywords:** physical activity, hemodialysis, body composition, myokines

## Abstract

*Background and Objectives*: The aim of this study was to assess the relationship between habitual physical activity, body composition, serum myokine concentration, and all-cause mortality in chronic hemodialysis patients. *Materials and Methods*: A prospective cohort study with a 7-year follow-up was conducted in a group of 38 patients (24 men, 14 women, mean age 65.6 ± 13.9 years, dialysis vintage 1.17 ± 1.25 years). Baseline serum concentrations of myokines—follistatin and myostatin—were assessed along with a measurement of physical activity with multidimensional accelerometery, body composition, and the force of forearm muscle contraction. Survival analysis was performed using the Kaplan–Meier method for tertiles of follistatin, serum myostatin, body composition, and physical activity expressed in metabolic equivalents (MET). *Results*: The mean physical activity among patients was 81 min/24 h (median 38.5 min), and the mean weekly 3MET activity was 493 min (median 218 min). The probability of survival of patients was significantly lower in the subgroup with 3MET/24 h less than 26 min/24 h and 3METt less than 148 min per week compared to the other subgroup (*p* = 0.006 and *p* = 0.006, respectively). During the 70-month follow-up, the subgroup with the lowest baseline follistatin concentration showed a significantly lower risk of death (*p* = 0.02). Baseline myostatin levels were not significant risk factors for mortality, nor were BMI or lean and fat tissue index categories. *Conclusions*: Physical activity and low plasma follistatin, but not body composition indexes or plasma myostatin, could serve as predictors of all-cause mortality in hemodialysis patients.

## 1. Introduction

Chronic kidney disease currently affects more than 10% of the world’s population, with an increasing trend. A low quality of life, numerous comorbidities, high mortality, and high risk of hospitalization among hemodialysis patients are common [1,2]. Patients undergoing hemodialysis are usually older and struggle with numerous chronic diseases.

In the hemodialysis population, most conventional risk factors of survival and the risk of cardiovascular events show an opposite relationship to that observed in the population with normal kidney function. Numerous studies published so far have suggested that, among hemodialysis patients, the occurrence of the classic and most serious risk factors for cardiovascular events, such as arterial hypertension, hypercholesterolemia, or a high body mass index and other parameters of nutritional status, do not necessarily increase the risk of cardiovascular events and death; in fact, even the paradoxical protective effect of the above-mentioned factors was suggested [3,4]. Although its existence is debatable, this phenomenon is commonly referred to as “reverse epidemiology” [5,6,7]. However, there are no clear data on whether a similar relationship applies to physical activity and biochemical indicators of skeletal muscle function.

Chronic hemodialysis patients are at high risk of developing malnutrition during renal therapy. Protein-energy wasting (PEW) is a significant risk factor for death in this population. [5] It is caused mostly by a decreased appetite and insufficient calorie intake, a catabolic–anabolic imbalance, chronic inflammation, the loss of nutrients during dialysis, and endocrine disorders or chronic metabolic acidosis. PEW manifests as a profound decrease in body mass, especially the fat component, a decrease in plasma albumin and lipids, and a loss of muscle mass. It may lead to the progression of cardiovascular disease due to accelerated atherosclerosis, which increases the risk of hospitalization and death and deteriorates the quality of life [8].

Body mass index (BMI) is calculated based on body weight and height. It categorizes people into groups of normal weight, underweight, overweight, and obese. BMI is easy to calculate and monitor but has numerous disadvantages as a clinical parameter. The body mass index does not refer in any way to the distribution of body weight between fat and lean body mass or the location of fatty tissue. It is not possible to determine a patient’s nutritional status based on BMI alone [9,10]. Hemodialysis patients constitute a distinct population in which an increased body mass is a direct consequence of sodium and water retention due to impaired or absent renal disposal. Therefore, fluctuations in body weight measurements are observed depending on the dialysis regimen. Numerous studies have described a lower risk of death among hemodialysis patients with a higher BMI, including values considered overweight and even obese [11]. BMI results may be inaccurate in the case of people with a disproportionately large muscle mass, as in the case of athletes, especially those practicing strength sports, or, on the contrary, among people with muscle atrophy. Due to the potential reduction in muscle mass in the course of sacropenia and the reduction in both fat and lean body mass in the course of PEW, the routine use of BMI in the context of hemodialysis patients and a comparison of values with those established for the general population may be subject to error.

Sarcopenia is a condition involving the loss of muscle mass and strength secondary to age and chronic disease. During hemodialysis therapy, some patients experience a progressive loss of muscle mass, with the subsequent weakening of muscle strength and decreased exercise tolerance. This reduction in muscle mass is accompanied by a decrease in quality of life, the development of disability, and an increase in the risk of death [12]. The pathomechanism of chronic sarcopenia in hemodialysis patients has not been precisely established, but it is believed that this disorder is mostly due to nutritional deficiencies, the loss of amino acids during the hemodialysis procedure, chronic inflammatory processes, vitamin D deficiency, hormonal alterations and uremia that lead to the predominance of protein degeneration processes over their synthesis [13]. Decreased muscle strength and mass are also a phenomenon occurring in the general elderly population, but their pathophysiological basis is different than that observed in dialysis patients [12].

Myostatin is a protein belonging to the transforming growth factor-β (TGF-β) family. It is produced and secreted primarily by skeletal muscles and, to a much lesser extent, by adipose tissue and cardiomyocytes. Myostatin adversely affects the proliferation and differentiation of skeletal muscle cells, limiting their growth through its regulatory effect on translational pathways [14]. Follistatin, which belongs to the same TGF-β family, is an inhibitor of myostatin that promotes muscle proliferation and growth [15].

Increased physical activity is a highly effective protective measure against cardiovascular disease in the general population. According to the 2021 guidelines of the European Society of Cardiology (ESC), for the primary prevention of cardiovascular diseases, it is recommended to undertake at least 150–300 min of moderate-intensity physical activity per week or 75 min of vigorous aerobic physical activity per week. Those who cannot follow recommendations because of physical limitations or chronic diseases are advised to perform any tolerated physical activity and avoid a sedentary lifestyle. The greatest benefits from increasing physical activity accrue to people with the lowest baseline activity [16]. The metabolic equivalent is used to objectively assess the intensity of physical exercise. One MET corresponds to the average energy expenditure while at rest. Moderate activity is 3–5.9 times the metabolic equivalent (MET).

According to the 2012 Kidney Disease Improving Global Outcomes (KDIGO) guideline, it is recommended to undertake 30 min of physical activity 5 times a week, although there are no specific recommendations regarding the type and intensity among different groups of patients depending on the stage of chronic kidney disease [17]. There are no worldwide clear recommendations regarding physical activity in chronic dialysis patients, as a population with a very high rate of sedentary lifestyles.

The United Kingdom Kidney Research Consortium Clinical Study Group for Exercise and Lifestyle published guidelines in 2022 for the exercise and lifestyle of patients with chronic kidney disease, including hemodialyzed patients. They recommend hemodialysis patients to strive for 150 min of moderate exercise per week or 75 min of vigorous physical activity per week or a mixture of both. This guideline also mentions intradialytic activity, which, according to the authors, is safe and has no contradictions [18].

Our study was designed to determine which of the parameters of body mass composition, including LTI and FTI, myostatin and follistatin, weekly and average daily physical activity, and the number of steps taken weekly and daily have a significant impact on the survival of chronic hemodialysis patients.

## 2. Materials and Methods

A prospective, single-center cohort study with a 7-year follow-up period was conducted. Ethical approval was obtained from the local ethical board (Permission Number: RNN/365/18/KE issued by the Ethics Committee of Medical University of Lodz on 13 November 2018). It included 38 patients undergoing chronic hemodialysis therapy (24 men and 14 women, mean age 63.6 years, median 66.5). The objective assessment of physical activity, body weight measurements with the assessment of fat and lean body mass, and the determination of concentrations of parameters was conducted using the following: the serum concentration of myostatin and follistatin, albumins, lipids, and CRP, which were performed from April to November 2016 after obtaining written consent from patients. The observation period lasted 7 years and ended in January 2023. The inclusion criteria included chronic hemodialysis for at least 6 months, three times weekly hemodialysis sessions with a Kt/V of at least 1.2. Exclusion criteria included a history of organ transplantation, malignancy, acute and chronic inflammation, treatment-resistant hypertension, class III and IV heart failure according to the New York Heart Association classification, diseases of the musculoskeletal or nervous system leading to the significant limitation of physical activity, liver disease, and intermittent hemodiafiltration or extended hemodialysis therapy.

Blood samples were collected immediately before the hemodialysis procedure and during the second session of the week in a fasting state. The blood sample was centrifuged at 2000 rpm at a temperature of 2–8 degrees °C, and plasma was later stored at −70 degrees °C until the tests were performed. The concentration of follistatin and myostatin in the serum was measured using an immunoassay and the Quantikine ELISA myostatin and follistatin kit manufactured by RnD Systems, Inc., Minneapolis, MN, USA. Follistatin and myostatin concentrations were determined once. The serum concentration of albumins, lipids, and CRP were measured using standard laboratory methods.

Body mass composition was assessed with multifrequency bioelectrical impedance using the Body Composition Monitor BCM (Fresenius Medical Care, Bad Hamburg, Germany, software version 3.3.0.1637). Measurements were performed after the second session of hemodialysis of the week. Patients were asked to place themselves in the supine position; after that, electrodes were placed on the hand and foot on the same side of the body. In patients with an arteriovenous fistula, the electrodes were placed on the side without a fistula. Obtained data were used to calculate lean tissue mass (LTM) and fat tissue mass (FTM) using the method described by Chamney et al. [19] and were transformed into index values relating them to the body surface area, obtaining the lean tissue index (LTI; LTM/height 2) and fat tissue index (FTI; FTM/height 2).

Physical activity was directly measured using a multidimensional accelerometery (BodyMedia SenseWear^®^ System version 7.0.0.2378; BodyMedia, Pittsburgh, PA, USA). After collecting blood samples, the study participants began a 7-day-long period of physical activity assessment. The accelerometer was worn on the trigeminal muscle of the non-dominant arm for continuous recording. Participants might have only removed the device when bathing or performing water activities. Patients were asked to maintain normal physical activity without trying to increase exercise for the duration of the measurement. After seven days of continuous measurement, participants were asked to return the equipment, and the physical activity data were analyzed using the dedicated software. The assessed parameters included the total and mean daily physical activity time measured as three times the metabolic equivalent (3MET), the total daily and weekly energy expenditure during physical activity (TEE), and the time spent at rest and sleeping. The force of the forearm muscle contraction was measured using a hand dynamometer on the dominant hand at the same time as the assessment of the body mass composition. We also measured skin folds using a caliper on the abdomen, triceps, and scapula.

After the measurements were completed, the patients were followed-up for the next seven years to analyze their survival time.

A descriptive analysis was performed, which included calculating the mean, median, and standard deviation values. Statistical calculations with survival analysis using the Kaplan–Meier method were performed for the tertiles of follistatin, myostatin, BMI, LTI, FTI, and physical activity parameters, including the total and mean daily physical activity time for at least 3MET and age. Calculations were performed using MedCalc software (MedCalc^®^ Statistical Software version 20.218 (MedCalc Software Ltd., Ostend, Belgium; https://www.medcalc.org, accessed on 20 April 2023).

## 3. Results

The baseline myokine concentration, measures of physical activity, the force of forearm muscle contraction, the time of physical activity measured by a metabolic equivalent (3MET), and the qualitative composition of body mass are presented in Table 1.

Thirty-seven percent of participants survived the 7-year follow-up. The mean survival time was 47.4 ± 27.4 months. The most common causes of death were infections (50%), but only one patient died because of COVID-19. Twenty-nine percent of patients died of cardiovascular incidents.

No differences were found in the analysis of the risk of death due to age in the study group. There was no correlation between the level of physical activity and age.

**Figure 1:**
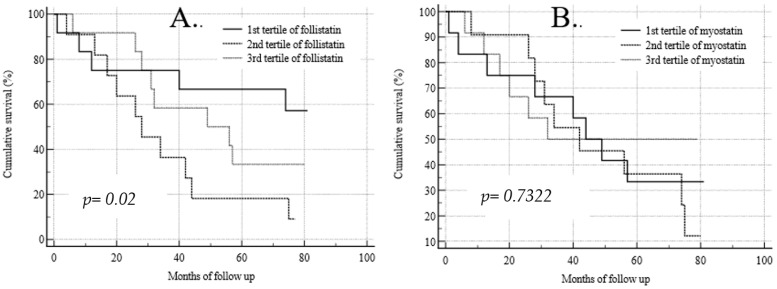
Kaplan–Meier analysis of survival for baseline follistatin (**A**) and myostatin (**B**) serum concentrations.

The Kaplan–Meier analysis of survival for the baseline follistatin and myostatin serum concentrations is shown in Figure 1. The survival analysis for the tertiles of baseline myostatin concentrations showed no significant differences between the tertiles with low, medium, and high serum myokine concentrations (*p* = 0.7322) (Figure 1B), unlike the tertiles of follistatin. During the 70-month follow-up, participants with the lowest initial follistatin concentration (mean 2271 ± 848 pg/mL) had a significantly lower risk of death than those with its medium concentration (4638 ± 796 pg/mL; *p* = 0.02) (Figure 1A). No such differences were observed during the full time of observation.

Participants wore the armband for an average of 97.3% of the time during the measurement.

**Figure 2:**
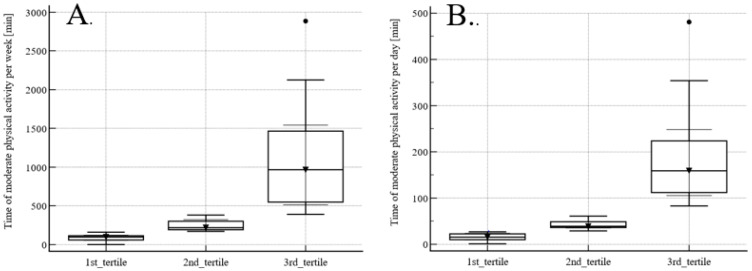
Tertiles of weekly (**A**) and daily (**B**) physical activity measured as 3MET.

The mean time of moderate physical activity (3MET) was 81 ± 105 min/24 h (median 38.5 min) and weekly 492 ± 502 min/7 days (median 218 min). The tertiles of physical activity are presented in Figure 2. The first tertile includes participants with the lowest (less than 27 min of 3MET daily), the second tertile with the medium (between 29 and 61 min of 3MET daily), and the third with the highest (more than 83 min daily) physical activity (Figure 2A). The first tertile includes participants with the lowest (less than 161 min of 3MET weekly), the second tertile with the medium (between 171 and 382 min of 3MET weekly), and the third with the highest (more than 391 min of 3MET weekly) physical activity (Figure 2B).

**Figure 3:**
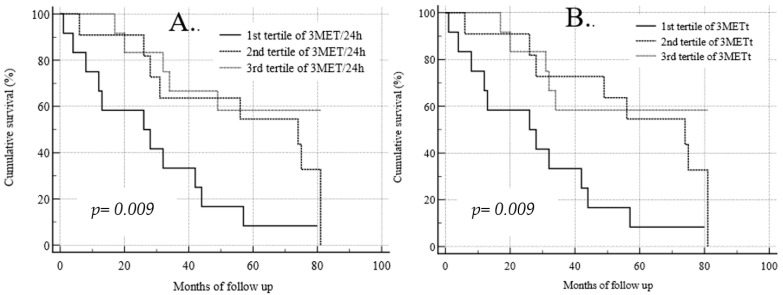
Kaplan–Meier analysis of survival for daily (**A**) and weekly (**B**) physical activity measured as 3MET.

The Kaplan–Meier analysis of survival for daily physical activity measured as 3MET is shown in Figure 3A. The probability of survival in the subgroup showing the lowest total physical activity measured as 3MET below 161 ± 47.1 min/7 days (thick line) was significantly lower (*p* = 0.009) than in subgroups with higher physical activity (dotted lines). The Kaplan–Meier analysis of survival for weekly physical activity measured as 3MET is shown in Figure 3B. Similarly, the risk of death was higher in the subgroup of patients with the lowest duration of moderate physical activity below 27 ± 8.6 min per day (thick line) compared to other subgroups presenting the medium and the highest measured daily physical activity (*p* = 0.009) with a mean duration of moderate daily physical activity of 61 ± 9.3 min/24 h and 481 ± 121 min/24 h, respectively (dotted lines).

The mean baseline contraction force was 25.6 kg ± 9.4 (median 24 kg), and the difference in the baseline contraction force was not a significant predictor of survival among the study participants. Only when the analysis period was limited to 70 months did study participants with the highest contraction strength, above 30 kg, have significantly higher survival compared to the remaining subjects (*p* = 0.0023; *p* < 0.001).

The total 7-day energy expenditure was 57,531 J ± 24,712 (median 59,416.5 J) and the total daily energy expenditure was 7191.4 J ± 3089 (median 7427.1 J). The total and mean daily energy expenditure during physical activity did not significantly predict survival.

The mean BMI among participants was 27.3 ± 4.4 kg/m^2^ (median 25.4 kg/m^2^). The mean LTI was 12.7 kg/m^2^ ± 4.5 (median 14.5 kg/m^2^), and the mean FTI was 9.1 kg/m^2^ ± 5.6 (median 5.5 kg/m^2^). There were no differences in survival between the tertiles of BMI, LTI, and FTI.

## 4. Discussion

A longer time of performing moderate physical activity, lower follistatin levels, and a stronger handgrip were associated with a lower risk of death from all causes among the participants of our study.

Patients treated with chronic hemodialysis are often older and struggle with numerous comorbidities, which significantly limits their ability to perform regular physical exercise. Therefore, in our study, we excluded patients with conditions that could significantly restrict physical activity, such as lung and cardiac disease, including severe heart insufficiency and musculoskeletal and nervous system diseases. Moreover, the participants were included after being on dialysis for at least half a year, thus avoiding the analysis-interfering effect of the increased early mortality seen in patients starting chronic dialysis therapy. At the same time, strict inclusion and exclusion criteria for the study limited the sample size, although it increased the homogeneity of our study population.

The 7-year observation period also covered the time of the COVID-19 pandemic, which is considered an unprecedented medical problem. According to the ERA-EDTA Registry, mortality due to COVID-19 infection in the group of dialysis patients was 20% higher than in the control group [20], with a decrease in the number of deaths from other causes at the same time [21]. However, among the participants in our study, only one person died due to COVID-19 infection, so the impact of the pandemic on our results seems to be negligible. A possible explanation for the lack of such interference is the relatively low age of our patients and the exclusion of subjects with advanced chronic diseases, including treatment-resistant hypertension and severe heart failure, which were significant risk factors for death among COVID-19 patients [21,22].

The determination of myostatin concentrations was performed once, without repeated measurements in the following years of observation and without determining the differences in concentrations before and after the hemodialysis procedure. Another study with multiple determinations of myokine concentrations should be performed in order to minimize the risk of accidental results.

Interestingly, unlike most previous studies [23,24], we did not observe any association between BMI and survival in our cohort of patients. This might be due to a limited cohort size or a narrow range of BMI in our group, with a mean BMI of 27.3 ± 4.4 kg/m^2^ (median 25.4 kg/m^2^). Only one patient had a BMI less than 18.5 kg/m^2^, which is known to be a risk factor for poor survival [25], and 8% had a BMI over 28 kg/m^2^, which is potentially favorable in the context of risk of death in a chronic hemodialysis population [26].

In recent years, there have been several large studies investigating physical activity levels among dialysis patients [27]. Most of them, especially those involving large groups of participants, were based on a subjective assessment of physical activity with questionnaires. Only several studies used objective measurements of physical activity for the analysis of survival over a period longer than 5 years, but they were conducted among an ethnically different population, i.e., in Japan [28,29]. However, these studies also confirm the role of higher physical activity as a potential protective factor in hemodialysis patients. Our study has limitations. First, it was conducted in a small group of patients from a single center, which may limit the generalizability of the findings to a larger population of chronic hemodialysis patients. All participants were of the same race, came from a limited area, and received medical care typical of the study center. The study group included mainly older people with a median age of a little over 65 years, which resulted from the demographics of the center conducting the study. Regardless of these reasons, limiting the study group to the elderly population reduces the possibility of translating the obtained results to the entire hemodialysis population. It can be speculated that hemodialysis patients from younger age groups might demonstrate higher physical activity, similar to the general population. An undoubted limitation of our study is the lack of analysis among the younger age group.

Furthermore, we only assessed the baseline, habitual physical activity, without taking into account a potential decline in activity during the follow-up, which could have occurred both as a result of the progressive deterioration of a general health condition in the course of long-term hemodialysis therapy or, secondary to the physiological aging process, the development of nervous, muscular, and skeletal diseases and accidental events causing a deterioration in patient’s general condition, such as injuries. On the other hand, the restrictive enrollment criteria applied in our study allowed us to create a group that was homogeneous in terms of its health condition and physical capabilities. Moreover, the survival analysis was performed based on a relatively long, 7-year observation period.

The assessment of physical activity was carried out using a direct measurement method in the form of multidimensional accelerometery, and the measurements were recorded for 7 days, including the days of hemodialysis sessions, when patients were forced to limit their physical activity during the procedure and often after it. This allowed us to assess real physical activity, thus limiting the risk of affecting the results by intentionally increasing the short-term activity of patients during the measurement time. Despite this, it cannot be ruled out that, during the measurement, participants intentionally undertook more physical activity than usual. The observation period of the full seven days seems to be a compromise between obtaining data covering different days of the week and periods of patient activity, including weekends, standard work days, dialysis and non-dialysis days, and at the same time, it is not too long, reducing compliance among subjects so as to not be excessively burdensome for them. The accelerometery measurement is non-invasive, easy to perform, and repeatable, but it does not take into account various types of activity, especially static activity such as cycling or lifting weights.

What seems interesting is the lack of significant statistical differences in the risk of death in relation to total and daily energy expenditure, while a difference in survival in relation to the level of physical activity was found.

Our patients remained at rest during the hemodialysis procedure. A recent study [30] showed the benefits of introducing exercise during hemodialysis treatments, which is consistent with the recommendations of physical activity created by the UK Kidney Research Consortium Clinical Study Group for Exercise and Lifestyle [18]

However, it should be remembered that a cause-and-effect relationship cannot be established based only on correlation.

## 5. Conclusions

The results of our cohort study show that a higher physical activity measured directly with an accelerometer with low plasma follistatin and a stronger handgrip was associated with a lower risk of death from all causes, which is consistent with the relationship seen in the general population [31,32] Therefore, the so-called “reverse epidemiology” phenomenon was not seen in the case of our analyzed population.

We did not observe a significant difference in the risk of death in relation to BMI, which was the same as the lean and fat tissue index. Despite survival differences between groups with different durations of moderate physical activity, we did not observe any differences in the total energy expenditure.

The results of our study are consistent with most previous studies that assessed the impact of physical activity on hemodialysis patients and confirm the validity of promoting increased daily physical activity in this group of patients. It seems reasonable to try to increase the physical activity of hemodialysis patients to the maximum tolerated value and to recommend avoiding a sedentary lifestyle.

It seems reasonable to strive to create global recommendations regarding specific lifestyle modifications, including clearly defined physical activity goals for the population of hemodialysis patients. According to Matsuzawa R et al., the suggested daily number of steps that may bring benefits in reducing the risk of death among dialysis patients is 4000 [29]. However, to validate this, further multi-center studies involving various nationalities and populations need to be conducted.

## Figures and Tables

**Table 1:** Mean and median baseline myokine serum concentration, weekly and daily physical activity, BMI, LTI, FTI and force of forearm muscles.

Parameters	Mean ± SD	Median
Miostatin (pg/mL)	2062.16 ± 975	1726.35
Folistatin (pg/mL)	7062.14 ± 4907	4756.5
3METt (min/7 days)	492.94 ± 444.7	218
BMI (kg/m^2^)	27.35 ± 3.3	25.4
FTI (kg/m^2^)	25.44 ± 3.6	14.5
LTI (kg/m^2^)	9.4 ± 4.3	9
Force of forearm muscles (kg)	27.35 ± 7.8	28

3METt—time of weekly physical activity measured as 3 times the metabolic equivalent of the task (moderate activity); 3MET/24 h—time of daily physical activity measured as 3 times the metabolic equivalent of the task (moderate activity); BMI—body mass index; FTI—fat tissue index; LTI—lean tissue index.

## Data Availability

The data presented in this study are available on request from the corresponding author.

## Abbreviations

3MET	3 times the metabolic equivalent
3METt	time of physical activity 3 times the metabolic equivalent per week
3MET/24 h	time of physical activity 3 times the metabolic equivalent per day
BMI	body mass index
COVID-19	Coronavirus disease 2019
CRP	C-reactive protein
ERA-EDTA	European Renal Association–European Dialysis and Transplantation Association
ESC	European Society of Cardiology
FTI	fat tissue index
FTM	fat tissue mass
J	Joule
KDIGO	Kidney Disease Improving Global Outcomes
KG	kilogram
LTI	lean tissue index
LTM	lean tissue mass
M	meter
MET	metabolic equivalent
PEW	protein-energy wasting
pg/m	Picograms Per Milliliter
TGF-β	transforming growth factor-β
TEE	total energy expenditure
